# Effectiveness of “Mom Supports Mom” Peer Support Intervention in Reducing Prenatal Anxiety and Psychosocial Stress Levels

**DOI:** 10.1192/j.eurpsy.2024.426

**Published:** 2024-08-27

**Authors:** K. Hrdličková, A. Horáková, M. Kuklová, H. Němcová, P. Knytl, L. Kostýlková, A. Šebela

**Affiliations:** ^1^National Institute of Mental Health, Klecany; ^2^Faculty of Arts, Department of Psychology; ^3^First Faculty of Medicine; ^4^Faculty of Science, Department of Demography and Geodemography; ^5^Second Faculty of Medicine, Department of Epidemiology; ^6^Third Faculty of Medicine, Charles University, Prague, Czech Republic

## Abstract

**Introduction:**

The prenatal period poses a risk of both onset and relapse of mental health difficulties. Anxiety and depressive symptoms are the most common, with a prevalence of 10-20%. Untreated mental health difficulties can have serious consequences for the child’s development, the quality of the mother-child relationship, and the whole family system. Peer support can be an effective form of care for women at risk.

**Objectives:**

The aim of the study is to examine the effectivness of remote *“Mom Supports Mom”* peer support intervention in reducing prenatal anxiety, depression and psychosocial stress levels.

**Methods:**

A randomized controlled trial was conducted. The Edinburg Postnatal Depression scale (EPDS) was used to assess the risk of mental health difficulties in pregnant women. Women with EPDS score ≥ 10 were randomized 1:1 to control and intervention groups. The intervention group received the *“Mom Supports Mom”* peer support intervention. The control group received care as usual. Between group-differences in anxiety, depression and psychosocial stress levels were measured one month after the enrollment/the start of the intervention. The Perinatal Anxiety Screening Scale (PASS), the Edinburg Postnatal Depression Scale (EPDS), and the Prenatal Psychosocial Profile (PPP) were used to assess the mental health difficulties.

**Results:**

The study involved a total of 67 participants in the intervention group, and 77 participants in the control group. Levels of anxiety (U = 2016, P < 0.05) and psychosocial stress (U = 1862, P = 0.001) were significantly decreased in the intervention group, showing a medium effect size of the intervention (Cliff’s delta= -0.218 and -0.317, respectively). There was no significant difference in depression levels (U = 2288.5, P = 0.243; Cliff’s delta = -0.113); see Table 1.

**Table 1:** Between group differences in study outcomes (n=144)

Measuring instruments	Intervention group (n=67) Median (IQR)	Intervention group 95% Confidence Interval		Control group (n=77) Median (IQR)	Control group 95% Confidence Interval		U value / t value	p-value
**EPDS (pre)**	13 (4)	12.5	13.9	13 (4)	12.8	14.2	2429.5	0.545
**PASS (pre)**	36 (17)	32.0	38.0	36 (15)	34.0	39.5	0.846	0.399
**PPP (pre)**	18 (4)	17.8	20.3	17 (6)	16.9	18.8	2282.5	0.233
**EPDS (dif )**	-6 (6)	-6.7	-4.1	-5 (6)	-5.4	-3.0	2288.5	0.243
**PASS (dif )**	-7 (11)	-12.3	-6.8	-5 (13)	-7.3	-2.4	2016	**0.024**
**PPP (dif )**	-2 (4)	-4.0	-1.6	0 (4)	-1.2	0.5	1862	**0.001**

EPDS = Edinburg Postnatal Depression Scale

PASS = Perinatal Anxiety Screening Scale

PPP = Prenatal Psychosocial Profile

pre = administered at baseline

dif = administered post-intervention

IQR = interquartile range

**Conclusions:**

The remote *“Mom Supports Mom”* peer support intervention can be effective in reducing anxiety and psychosocial stress levels in at-risk pregnant women. Nevertheless, it didn’t show effectivness in reducing depression levels.

**Disclosure of Interest:**

None Declared

